# Mucinous differentiation features associated with hormonal escape in a human prostate cancer xenograft

**DOI:** 10.1038/sj.bjc.6601570

**Published:** 2004-02-03

**Authors:** M-E Legrier, G de Pinieux, K Boyé, F Arvelo, J-G Judde, J-J Fontaine, J Bara, M-F Poupon

**Affiliations:** 1 Institut Curie, Section de Recherche, FRE2584 CNRS, France; 2 Hôpital Cochin, Anatomie Pathologique, Paris, France; 3 Institut Curie, Section Médicale, 26 rue d’Ulm, Paris 75248, France; 4 Ecole Nationale Vétérinaire, Service d’Anatomie Pathologique, 7 av du Général de Gaulle, Maisons Alfort cedex 94704, France; 5 Hôpital Saint-Antoine, U-482 INSERM, 184 rue du Faubourg St-Antoine, Paris 75012, France

**Keywords:** prostate cancer, hormone independence, morphological differentiations, mucins

## Abstract

Many theories mention hypersensitive, promiscuous, outlaw or bypass signalling pathways to explain the acquisition of hormone independence in prostate cancer. Hormonal escape of prostate tumours is marked by many biological changes, including mucinous and neuroendocrine differentiation. Since expression of several mucins has been linked to carcinoma tumour progression, we have characterised the expression of mucins at both RNA and protein levels in an *in vivo* model of prostate cancer in hormonal escape. Using PAC120, a xenograft of a human hormone-dependent prostate tumour, and its hormone-independent variants, we analysed the expression of mucins (MUC1, MUC2, MUC4, MUC5AC, MUC5B, MUC6) by immunohistochemistry or reverse transcriptase (RT)–PCR. While the parental PAC120 tumour was a compact poorly-differentiated tumour of Gleason score 9 (5+4), hormone-independent variants displayed mucinous, neuroendocrine-like or mixed histological changes; these changes were stable through serial transplantations or after testosterone supply. MUC1 mRNA was expressed in both PAC120 and the hormone-independent variants, although at variable levels. All tumours displayed a high and constant expression of MUC2 and no expression of MUC4 mRNA. While MUC1 was expressed in all xenografts whatever their hormone dependence status, MUC2, MUC5B and MUC6 were preferentially expressed in hormone-independent variants. The loss of hormone dependence in this prostate cancer xenograft model is therefore marked by irreversible histological alterations, mucinous or neuro-endocrine, associated with an expression of secretory MUC2, MUC5B and MUC6, independent of the histological differentiation subtype. These data point to mucinous differentiation as an important step in the acquisition of hormone independence in this cancer, and suggest that secretory mucins might participate in an unknown pathway of hormonal escape in prostate cancer.

Androgen deprivation therapy has been used for decades in the treatment of prostate cancer (Huggins, 1967). However, although this treatment is initially very effective in these hormone-dependent cancers, they invariably become hormone-refractory and metastasise, leading to the death of patients. Recently, Feldman and Feldman (2001) reviewed possible mechanisms by which prostate cancer can escape androgen deprivation therapy. While the androgen receptor (AR) is activated by testosterone-induced phosphorylation in hormone-dependent prostate tumours, hormone-independent growth might be due to the activation of AR via a phosphorylation event induced by other growth factor receptors, such as HER1 (EGFR) and HER2 (Her2/neu) (Kwong and Hung, 1998; Yeh *et al*, 1999). In addition, several recent studies have linked the IGF-1 receptor pathway to the stimulation of the androgen-signalling pathway in hormone-refractory prostate cancer (Culig *et al*, 1994; Bubendorf *et al*, 1999). Hormone-independent growth of prostate tumours has also been associated with biological changes such as mucinous and neuroendocrine differentiation. Neuroendocrine differentiation has been observed in prostate cancer and has been correlated with tumoural aggressiveness, short survival and poor response to endocrine therapy (McWilliam *et al*, 1997), and was considered to be an early marker of progression toward hormone independence (Cohen *et al*, 1991; Di sant’Agnese and Cockett, 1996; Noordzij *et al*, 1996). Although true mucinous or colloid prostatic adenocarcinoma, with extensive mucin production, remains a rare entity (Epstein and Lieberman, 1985; Nagakura *et al*, 1986), production of neutral mucin as assessed by immunohistochemistry is found in up to 55% of prostate carcinomas (Sentinelli, 1993). A total of 19 different mucin genes (MUC1–MUC4, MUC5B, MUC5AC, MUC6–MUC18) have been identified to date and divided into two groups: those coding for membranous mucins such as MUC1, MUC3 and MUC4, and those coding for secreted mucins such as MUC2, MUC5AC, MUC5B and MUC6. Secreted mucins are glycoproteins constituting the major macromolecular component of mucus, while membrane-associated mucins contribute to epithelial cell–cell interactions. Their pattern of expression, especially for the secreted mucins, appears to be relatively tissue-specific. However, the distribution and type of mucin produced by normal prostatic tissue and prostatic carcinomas are not well documented (Daher *et al*, 1990), though focal mucin production in conventional prostatic adenocarcinomas has been recognised for many years (Pinder and McMahon, 1990). Mucin expression has also been observed in a few cases of prostatic intraepithelial neoplasia (PIN) (Sentinelli, 1993).

MUC1, designated also as the CA19.9 marker, is frequently expressed in many types of cancer (Chu and Chang, 1999), and was found to be highly expressed in normal prostatic glandular tissue and in prostatic adenocarcinoma (Ho *et al*, 1993). Its detection in the blood is a good indicator of the presence and burden of the tumour, but its biological role in cancer is poorly understood. In a recent study, its expression was detected in 94% of prostate tumours examined, and the intensity of the cytoplasm staining was significantly correlated with the tumour grade and stage (Kirschenbaum *et al*, 1999). MUC2 is not expressed in any prostatic tissues (Ho *et al*, 1993), but in the majority of colon adenocarcinomas, particularly those of colloid type (Hanski *et al*, 1997). MUC5AC is highly expressed in normal prostatic glandular tissue (Daher *et al*, 1990) and in colon adenocarcinomas (Bara *et al*, 1991), while MUC4, MUC5B and MUC6 are not expressed in prostatic glandular tissue.

In this study, we have used a xenograft model of human prostate cancer to explore the changes in histology and mucin expression patterns occurring during the progression to hormone independence. PAC120 is a human prostate cancer xenograft obtained by urethral resection of a recurrent prostate cancer, and direct subcutaneous engraftment into nude mice (de Pinieux *et al*, 2001). PAC120 is androgen-responsive, but recurs as hormone-refractory disease following transplantation in castrated mice. We have compared the hormone-responsive PAC120 parental tumour with several independently obtained hormone-refractory tumour variants for their mucinous and neuroendocrine histological components, and analysed the relationship between these phenotypic changes and the expression of mucins at the RNA and protein levels.

## MATERIALS AND METHODS

### Prostate tumour xenografts

All the experiments were realised *in vivo* and have been carried out with ethical committee approval, and meet the standards required by the UKCCR guidelines (Workman *et al*, 1998).

The parental tumour PAC120, a hormone-dependent human prostate cancer xenograft transplantable into nude mice, was established in our laboratory (de Pinieux *et al*, 2001). The original PAC120 tumour appeared 7 months after grafting, and was maintained by serial transplantation during several passages (p4–p29) by subcutaneous implantation of tumour fragments. The mean delays between two passages were of 5 months. HIDs are hormone-independent variants that were obtained in nude mice after surgical castration. PAC120-bearing mice were castrated when the local tumours reached 250–500 mm^3^. After a variable latency, HID tumours started to grow again. Seven HID variants were independently isolated. The recurrence latencies were 16, 7, 8 months for HID25, HID28, HID33, respectively, and 12 months for the four later variants HID16, HID19, HID34 and HID35. Delays before the second passage in precastrated males were between 3 and 6 months. One variant, HID28, was transplanted into both castrated and uncastrated male mice.

### Immunochemical studies

Two PAC120 tumour samples (passages p4 and p20) and the seven HID tumours were removed from mice and immediately fixed in a 95% ethanol solution for immunohistological studies of MUC1, MUC2, MUC5AC (Daher *et al*, 1990), MUC5B and MUC6 expression; this fixation is known to protect the integrity of mucin epitopes. All tumour samples were screened for mucin production with alcian blue (pH 2.5) and periodic acid Schiff stains. The antibodies used for immunohistochemical (IHC) studies, their sources and dilutions are listed in Table 1

**Table 1:** Immunoreagents used in the immunohistochemical analysis

**Reagent**	**Source**	**Dilution**	**Positive control**
Anti-MUC1 (monoclonal H23)	Transgene (Strasbourg, France)	1 : 100	Breast cancer
Anti-MUC1 (monoclonal M8)	A gift from S Gendler (Gendler and Spicer, 1995)	1 : 10	Gastric mucosa
Anti-MUC2 (polyclonal Lum 2–3)	I Carstedt	1 : 1000	Colonic mucosa
Anti-MUC5B (monoclonal EU1)	D Swallow	1 : 10	Bronchial epithelium
Anti-MUC5B (polyclonal Lum 5B-2)	I Carstedt	1 : 1000	
Anti-MUC5AC (monoclonal 21M1)	A gift from J Bara (Daher *et al*, 1990)	1 : 10	Gastric mucosa
Anti-MUC6 (monoclonal F8)	P Real	1 : 10	Gastric mucosa

. Tissues employed as positive controls are indicated in the same table.

Standard immunohistochemistry by avidin–biotin complex (ABC) immunoperoxidase technique was done as follows. Paraffin-embedded sections, 4-*μ*m thick, were used for light microscopy examination after haematoxylin–eosin–safran (HES) staining, and for immunohistochemistry. Immunohistochemical study of MUC1, MUC2, MUC5B, MUC5AC and MUC6 mucin expression was performed on all tumour samples.

Immunostaining was performed on sections, mounted onto silane coated slides, air dried, deparaffinised in xylene and rehydrated. Slides were washed three times in phosphate-buffered saline (PBS, pH 7.4) between each incubation step of the procedures. Endogenous peroxidase activity was blocked by incubation of samples in 0.3% hydrogen peroxide in methanol for 30 min. Tissue slides were microwaved in 0.01 M sodium citrate buffer (pH 6) near boiling for 20 min, and cooled for 30 min in the buffer before incubation with the primary antibodies. Sections were then incubated with normal serum (1 : 20 in PBS) for 20 min to block nonspecific serum-binding sites. Primary antibodies were incubated on tissue sections at room temperature for 1 h. After incubation with a biotinylated secondary antibody, the immunohistochemical reaction was visualised using ABC (Vectastain Elite kit, Vector Laboratories, Burlingame, CA, USA) with the chromogen amino-ethylcarbazol (AEC). Sections were counterstained by Mayer's haematein solution. In each case, appropriate positive and negative controls were tested simultaneously. Staining intensity was assessed semiquantitatively as 0 (negative), +(weak to moderate) and ++(strong). The pattern distribution of staining was focal (F) or diffuse (D).

### Detection of mRNA transcripts by reverse transcriptase (RT)–PCR

Xenograft samples were harvested 6–8 weeks after grafting and snap-frozen in liquid nitrogen for subsequent RNA extraction. RNA was prepared using a commercially available kit (Trizol, Invitrogen, Gercy Pontoise, France). RNA quality was confirmed by gel electrophoresis and ethidium bromide staining, or by RNA 6000 Assay (Agilent Technologies 2100 Bioanalyser, Massy, France). Reverse transcription of RNA was performed in a final volume of 20 *μ*l containing 5 × RT buffer (Invitrogen), 100 mM DTT, 200 ng ml^−1^ oligodT, 2.5 mM dNTP and 200 units l^−1^ of reverse transcriptase. The samples were incubated at 42°C for 1 h, and then kept frozen until use. A measure of 100 ng of cDNA was used for each PCR reaction. PCR amplification was performed using human tubulin-*β*2 primers as control, and MUC1, MUC2 and MUC4 primers (Table 2

**Table 2:** Primers used for the study of mucin genes by RT–PCR

**Genes**	**Primers 5′ → 3′**	**Position**	**bp**	**GENBANK Accession no**
MUC1	S:ACTCTGATACTCCTACCACCCTTG	830–853	406	X52228
	AS:CACCCAGAACTGTACCTGAACTTA	1235–1212		
MUC2	5:CTGCACCAAGACCGTCCTCATG	15291–15312	401	L21998
	AS:GCAAGGACTGAACAAAGACTCAGAC	15667–15699		
MUC4	S:CGCGGTGGTGGAGGCGTTCTT	3094–3114	596	AJ24246
	AS:GAAGAATCCTGACAGCCTTCA	3670–3690		
Tubulin-*β*2	5:CGAAGCTCTCTACGACATT	669–688	489	NM 006088
	AS:GAAGGTGGCGGACATTTTTAG	1139–1158		

). The thermal cycling conditions comprised 40 cycles at 94°C for 1 min, 60°C for 1 min and 72°C for 1 min.

For real-time RT–PCR, RT of RNA was realised by random priming (cDNA cycle Kit, Invitrogen). Primers for the MUC1 mRNA were designed with the Primer Express (ABI, Les Ulysses, France) and ClustalX softwares. We conducted BLASTN searches to confirm the gene specificity of the nucleotide sequences chosen (Table 3

**Table 3:** Primers used for the study of MUC1 gene by real-time RT–PCR

**Genes**	**Primers 5′ → 3′**	**Position**	**bp**	**GENBANK Accession no**
HPRT	S: GCTTTCCTTGGTCAGGCAGTATAA	523–546	141	NM 000194
	AS: AAGGGCATATCCTACAACAAACTT	641–664		
MUC1	S: CTTTCTTCCTGCTGCTGCTCCT	22–44	95	NM 002456
	AS: AGCCGAAGTCTCCTTTTCTCCA	96–117		

). To avoid amplification of contaminating genomic DNA, one of the two primers was placed across a splice junction. QPCR was performed using the qPCR™ Core Kit for Sybr™ Green I (Eurogentec, Seraing, Belgium) to quantify the MUC1 transcripts. DNAc (50 ng) was used for each PCR. The thermal cycling conditions comprised an initial denaturation step at 95°C for 10 min and 45 cycles at 95°C for 15 s and 60°C for 1 min. Experiments were performed in triplicate for each data point. We quantified HPRT transcripts (human hypoxanthine phosphoribosyltransferase) as an endogenous RNA control, and each sample was normalised on the basis of its HPRT content. Biologically significant variations were defined for genes with a ratio ⩾2.

## RESULTS

### Histological features of PAC120 and HID tumour xenografts

**Figure 1:**
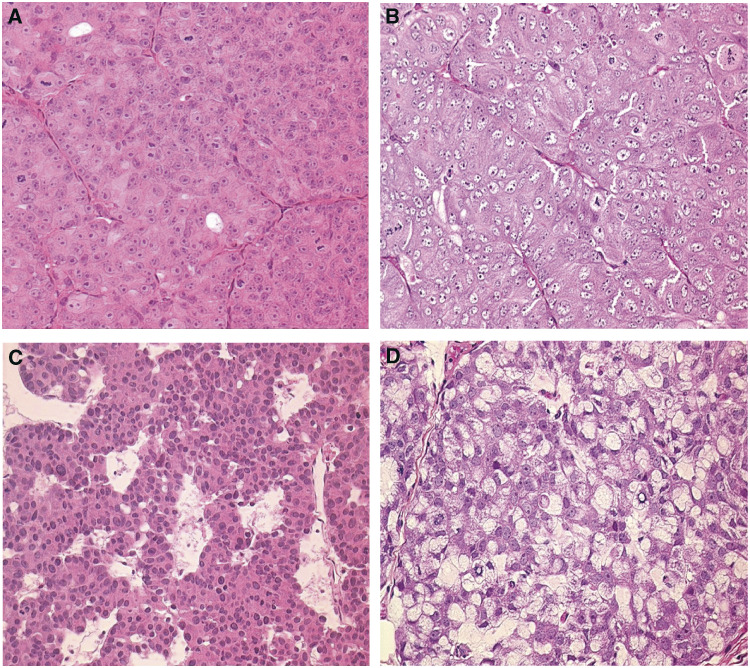
Histology of human prostate cancer after xenografting, PAC120 and HID variants. (**A**) PAC120, compact with glandular differentiation (HES × 200). (**B**) HID28, a microscopic pattern similar to that of the PAC120 tumour (HES × 200). (**C**) HID16, focal neuroendocrine-like pattern (HES × 200). (**D**) HID25, a fully mucoid adenocarcinoma (HES × 200).

The histological features of PAC120 xenografts were those of a poorly differentiated, Gleason score 9 (5+4), conventional prostate adenocarcinoma, without any evidence of mucus secretion. PAC120 reproduced the morphological and biological characteristics of the original tumour as published (de Pinieux *et al*, 2001), with a compact, lobular pattern and sparse gland lumens (Figure 1A

). During serial transplantation, PAC120 displayed a very stable morphology and histology (neither mucinous nor neuroendocrine differentiation were observed), and remained hormone-dependent for 29 passages over 7 years.

Castration of tumour-bearing hosts induced tumour growth arrest, followed by a decrease in tumour size. Tumours recurred after various prolonged delays, from 7 to 16 months. The recurrent tumours kept growing upon transplantation into castrated mice, confirming their hormone independence. Seven hormone-independent variants (HID) were obtained independently from PAC120. Compared to the parental hormone-dependent PAC120 tumour, HIDs displayed distinctive histological changes typical of advanced prostate cancer, such as mucinous, neuroendocrine-like or mixed differentiation features. Several tumours originating from the same variants were studied. The histological pattern of HID28 (Figure 1B) and HID16 was close to that of PAC120, with few areas exhibiting a neuroendocrine-type pattern (Figure 1C) or mucinous areas. Growth of HID28 tumours in intact animals did not alter tumour morphology, although their growth was accelerated (de Pinieux *et al*, 2001). HID25 contained principally signet-ring cells (Figure 1D), while HID33 was mucinous. HID19 consisted of a clear-cut juxtaposition of areas of compact poorly differentiated carcinoma and mucinous adenocarcinoma. In HID19, the less-differentiated tumoural component showed pleiomorphic tumour cells arranged in large sheets, without glandular differentiation. HID34 and HID35 tumours exhibited focal areas, with a morphological pattern associating neuroendocrine-type with mucinous areas. The histological patterns of these different tumour variants are summarised in Table 4

**Table 4:** Expression patterns of mucins in xenografts of PAC120 prostate adenocarcinoma and of its HID variants (Immunohistochemistry detection)

**Tumours**	**Histological pattern**	**MUC1**	**MUC2**	**MUC5AC**	**MUC5B**	**MUC6**
PAC120	Compact area	++^a^^,^^c^	+^b^^,^^c^	+^b^^,^^c^	0	0
	Mixed^d^					
HID16	Compact area	++^a^^,^^c^	+^b^^,^^c^	0	0	0
	Neuroendocrine area	++^a^^,^^c^	0	0	0	0
						
*Mixed:*						
HID28	Compact area	+^b^^,^^e^	+^b^^,^^e^	0	+^b^^,^^e^	0
	Neuroendocrine area	0	++^f^^,^^g^	0	+^g^	++^g^
HID25	Mucinous area	+^a^^,^^e^	++^f^^,^^g^	0	+^g^	++^g^
HID33	Mucinous area	++^a^^,^^c^	+^b^^,^^c^	0	0	0
						
*Mixed:*						
HID19	Compact area	++^a^^,^^c^	0	0	0	0
	Mucinous area	+^a^^,^^e^	++^f^^,^^g^	0	+^g^	++^g^
						
HID34	*Mixed:*					
HID35	Neuroendocrine area	++^a^^,^^c^	0	0	0	0
	Mucinous area	0	++^f^^,^^g^	0	+^g^	++^g^

Staining intensity: 0: negative; +: low to moderate; ++: strong. Immunostaining:

a focal

b on rare cells;

c cytoplasmic;

d tumour samples exhibiting different patterns of differentiation with or without testosterone support;

e membranous;

f signet-ring cells;

g extracellular mucin lakes.

**Figure 2:**
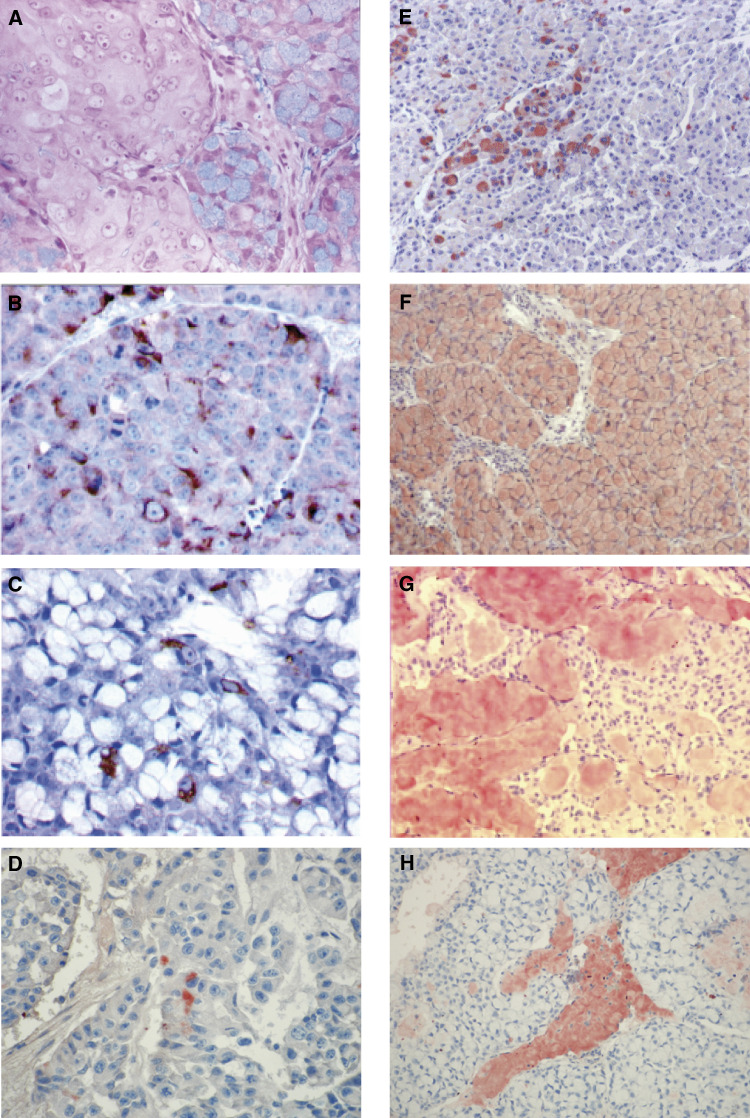
Expression patterns of different mucins in xenografts PAC120 and HID variants by immunohistochemistry. (**A**) Alcian blue pH 2.5 in HID-19: signet-ring cells staining positively (HES × 400) (**B**) MUC1 expression in PAC120: cytoplasmic immunostaining in a fraction of the tumour cells (HES × 400). (**C**) MUC1 expression in HID25: scattered tumour cells staining positively for MUC1 with a cytoplasmic pattern (HES × 400). (**D**) MUC2 expression in PAC120 p4: rare tumour cells exhibiting cytoplasmic staining (HES × 400). (**E**) MUC2 expression in HID16: rare tumour cells exhibiting cytoplasmic staining (HES × 100). (**F**) MUC2 expression in HID25: uniform and strong intracytoplasmic positivity of signet-ring cells (HES × 100). (**G**) MUC6 expression in HID35, mucinous areas: strong staining of extracellular mucin lakes (HES × 100). (**H**) MUC5B expression in HID19, mucinous areas: strong staining of extra cellular mucin lakes (HES × 100).

. Four of the seven HID variants presented a dominant neuroendocrine differentiation and five a dominant mucinous phenotype. In all cases, the mucinous tumours contained signet-ring cells, extracellular mucin lakes (colloid carcinoma areas) and glandular structures delimited by a layer of mucin-secreting tumour cells. Intra- and extracellular mucin stained positively for alcian blue pH 2.5 (Figure 2A

) and periodic acid Schiff.

### Immunohistochemical detection of mucins

MUC1 was detected in PAC120 tumours, the anti-MUC1 antibody staining focally the cytoplasm of 10–20% of tumour cells (Figure 2B). The anti-MUC2 and anti-MUC5AC antibodies stained the cytoplasm of very few tumour cells in the compact areas (data not shown). Anti-MUC5B and anti-MUC6 antibodies did not stain PAC120 tumours (data not shown). It should be emphasised that staining of mucins by the selected antibodies was strictly dependent on the tissue-fixation method, as we used a 95% ethanol solution, a technical published by one of us (Daher *et al*, 1990).

In the HID variants, MUC1 was expressed focally with cytoplasm positivity in HID16, HID19, HID33, or membrane positivity in HID19, HID25, HID28, and in the neuroendocrine-type tumour foci of HID16, HID34 and HID35. MUC1 immunoreactivity was observed at the apex of occasional tumour cells delimiting mucosecreting glandular tubes in HID25 (Figure 2C) and in HID19 tumours. MUC1 and MUC2 mucins were not simultaneously expressed in tumour cells. Anti-MUC2 antibodies stained rare tumour cells in PAC120 p4 (Figure 2D) and in HID16 (Figure 2E), HID28 and HID33, while signet-ring cells were uniformly stained in HID25 (Figure 2F) and in the other HID variants. Extracellular mucin lakes were strongly stained by the anti-MUC2 and anti-MUC6 antibodies (Figure 2G), and weakly by the anti-MUC5B antibodies (Figure 2H). MUC5AC mucin was not expressed in the HID xenografts. There was no significant modification in mucin expression between HID28 tumours with or without testosterone supply. Colorectal mucosa was used as positive controls for MUC2, gastric mucosa for MUC1, MUC5AC, MUC6 and bronchial mucosa for MUC5B (Table 1). MUC2 was not detected in normal prostate tissue, except in cells derived from the duct utriculum (data not shown). The immunohistochemical localisation of different mucins is reported in Table 4.

### Evaluation of mRNA transcript levels by RT–PCR

**Figure 3:**
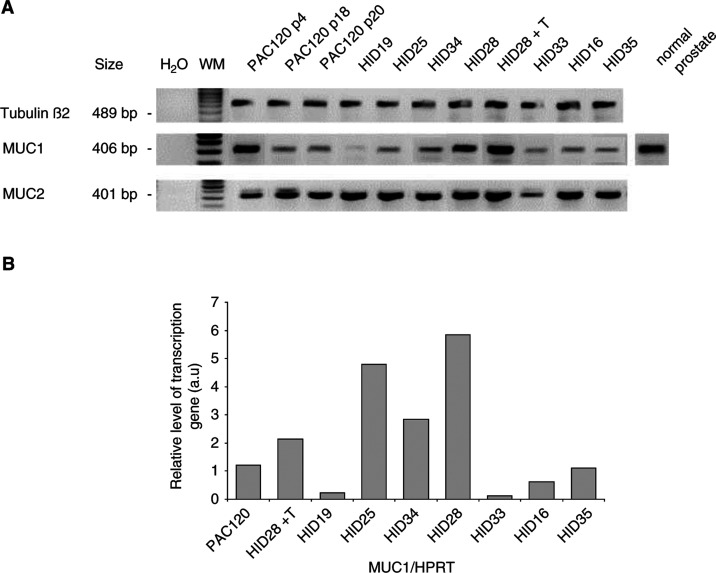
RT–PCR analysis of mucin genes expression in PAC120 and HID variants. (**A**) mRNA transcripts of human MUC1, MUC2 and tubulin-*β*2 genes by RT–PCR. mRNA was extracted from hormone-dependent and -independent tumours. Products of RT–PCR of PAC120, HID variants and normal prostate tissue were deposited successively. (**B**) Expression of MUC1 transcripts by real-time PCR into the different samples, compared to HPRT gene. Products of RT–PCR of PAC120 and HID variants were deposited successively.

Evaluation of the expression of the mucin genes at the RNA level was done in the same samples that were used for immunohistochemistry, and in samples derived from successive transplantations. Expression of the different mucins was investigated by semiquantitative RT–PCR using oligonucleotides specific for human MUC1, MUC2 and MUC4 (Figure 3A

). Expression of tubulin-*β*2 was used as endogenous control. The MUC1 transcript was detected at low levels in HID16, HID19, HID33 and HID35 and at higher levels in the other variants, while MUC2 was expressed at high levels in PAC120 and in all HID variants. No MUC4 expression was detected, while a small-cell lung tumour (SCLC-6) used as control was positive. MUC1 mRNA expression was also assessed by real-time RT–PCR, using HPRT as the reference gene against which MUC1 mRNA levels were normalised (Figure 3B). Again, MUC1 expression was moderate to high in hormone-dependent xenografts, and variable in hormone-independent prostate xenografts, according to the semiquantitative RT–PCR results. Analysis of real-time PCR data showed that MUC1 expression decreased when HID28 grew in intact animals, that is, with testosterone support.

## DISCUSSION

Tumour escape from hormone deprivation therapy is a major obstacle in the clinical management of prostate cancer. However, the biological basis of the acquisition of hormone independence by these tumours is still poorly understood. This is in part due to the difficulty in obtaining clinical samples at the various stages of prostate tumour progression, and also to the fact that only a few experimental systems are amenable to the study of prostate cancer hormonal escape (Nagabhushan *et al*, 1996).

PAC120 is a hormone-dependent poorly differentiated prostate adenocarcinoma xenograft that displayed no major morphological changes along successive transplantations (passages p4–p29), and from which several distinct HID variants have been obtained *in vivo* (de Pinieux *et al*, 2001). It is noteworthy that the hormone-dependent phenotype of PAC120 was consistently maintained for 7 years. Morphological changes appeared as a consequence of hormonal escape after surgical castration. HID tumour variants presented three different phenotypes: neuroendocrine, mucinous or mixed. The neuroendocrine type was characterised by tumour cells arranged in large tumoural cords intermingled in a conjunctiva-vascular stroma. The mucinous differentiation associates glandular structures delimited by a layer of mucin-secreting tumour cells, extracellular mucin lakes and independent signet-ring cells. In HID variants, the neuroendocrine tumours presenting a variable contingent of neuroendocrine areas (four out of seven) and the mucinous variants (five out of seven) were frequent and stable during successive passages. Some variants presented as poorly differentiated adenocarcinomas, as observed in the parental PAC120 tumour.

The significance of these morphological changes remains unknown, though they must be somehow related to tissue suffering and survival as a consequence of hormone deprivation. Neuroendocrine and mucinous differentiation appeared in almost all HID variants, with a predominance of one phenotype but frequently mixed, as if a superior degree of tumour heterogeneity was reached by HID in the course of hormonal escape. In the present study, we focused on the analysis of the changes in the mucin expression pattern associated with the acquisition of hormone independence. Mucinous differentiation in adenocarcinomas is recognised as characteristic of advanced tumoural stage, especially in prostate and breast cancers. Production of focal mucus, detected by histochemistry, was observed in 43–61% of prostatic adenocarcinomas (Ro *et al*, 1988; Pinder and McMahon, 1990). A true mucinous differentiation, involving more than 50% of tumour cells, is rare and is a marker of poor prognosis (Pinder and McMahon, 1990). In colon and breast adenocarcinomas, expression of mucins was also associated with a low survival rate (Nakamori *et al*, 1994; McGuckin *et al*, 1995). Mucin expression may contribute to cancer cell survival during tumour progression and hypoxic conditions found at advanced tumour stages. Although mucinous differentiation has been observed in patients with advanced stage prostate cancer (Ro *et al*, 1988; Pinder and McMahon, 1990), this was the first observation of such changes being directly related to hormone deprivation and the acquisition of hormone independence.

Human mucin genes constitute a family of 19 glycoproteins, which can be divided into two groups: membrane-bound mucins (MUC1, MUC3 and MUC4) and secreted mucins (MUC2, MUC5AC, MUC5B and MUC6). The expression profile of mucins differs according to the glandular tissue type. Mucins are encoded by genes located on distinct chromosomes; MUC1, MUC3, MUC4, MUC7, MUC8 are located on chromosome 1, 7, 3, 4, 12, respectively, while MUC2, MUC5AC, MUC5B, MUC6 are clustered in chromosome 11p15. Expression of membrane-bound mucins MUC1 and MUC4 was analysed in our model. MUC1 mRNA was detected in PAC120 xenografts, although at variable levels and regardless of the morphological pattern. By immunohistochemistry, the expression profile of MUC1 in the hormone-independent poorly differentiated, neuroendocrine and mucosecreting subtypes was similar to that of hormone-dependent tumours. A focal and intracytoplasmic MUC1 immunostaining was observed in 10–20% of tumour cells. There was no quantitative relationship between the MUC1 mRNA and protein expression level. A hypothesis was that HID cells displayed an adaptation to the environmental conditions, which implicated the activation of some genes necessary to their survival during hormonal escape. MUC1 expression was found in most adenocarcinomas of the breast, lung, stomach, pancreas, prostate and ovary (Ho *et al*, 1993), and a diffuse cytoplasm MUC1 immunostaining was associated with a high Gleason score (Kirschenbaum *et al*, 1999). MUC4 mRNA, which was expressed in various epithelial tissues, was not expressed in PAC120. MUC4 was expressed particularly in lung carcinomas (Copin *et al*, 2001) and, interestingly, it was proposed as a ligand for HER2 (Moniaux *et al*, 1999; Carraway *et al*, 2002), a growth factor receptor of the tyrosine kinase family, putatively implicated in hormonal escape of breast and prostate cancers. Thus, in the present study, these membrane-bound mucins do not seem to be involved in the acquisition of hormone independence, since MUC4 was not expressed and MUC1 expression was not associated with any particular morphological pattern.

Actually, the biological role of MUC1 is unclear. It was suggested that MUC1, a mucin detected in an important proportion of prostate cancers (Kirschenbaum *et al*, 1999), was associated with a decrease of cell to cell (Ligtenberg *et al*, 1992) or cell to extracellular matrix (van de Wiel-van Kemenade *et al*, 1993; Wesseling *et al*, 1996) interactions, by masking cellular adhesion molecules. In addition, the cytoplasmic domain of MUC1 is involved in signalling pathways through its interaction with *β*-catenin (Li *et al*, 1998; Carraway *et al*, 2002), this interaction being dependent on their phosphorylation by the glycogen synthase kinase 3*β* (GSK3*β*) (Li *et al*, 1998; Pignatelli, 1998; Lickert *et al*, 2000). The variations of phosphorylation might induce variations of *β*-catenin affinity for its substrates and therefore its nuclear transfer. *β*-catenin significantly enhances androgen-stimulated transcriptional activation by the AR. The coimmunoprecipitation of *β*-catenin with AR from LnCaP cells showed that the two molecules are present in the same complex, this binding being dependent on androgen (Truica *et al*, 2000).

Regarding secreted mucins, MUC2 epitopes were not expressed in normal prostatic tissue (Ho *et al*, 1993) except near the utriculum, but were expressed in prostate cancer. Although the mRNA of MUC2 was expressed at constant levels in PAC120 and HID tumours, a variable expression of the MUC2 protein was observed. While only a few tumour cells of PAC120 expressed MUC2, the HID variants contained much more cells staining positive for MUC2. Such a discrepancy between mRNA and protein levels suggests that MUC2 expression is subject to differential post-transcriptional regulation in hormone-dependent (HD) and HID tumours. The mucosecreting tumoural subtypes presented a completely different pattern of mucin expression, including a high and predominant MUC2 expression. MUC2 expression was characterised by both an intracellular accumulation in signet-ring cells and an extracellular localisation in mucin lakes. This profile was similar to that of mucinous or colloid mucinous adenocarcinomas observed in advanced cancers of various origins, such as the gastrointestinal tract, ovary or breast (Hanski *et al*, 1997). This observation suggests that acquisition by tumour cells of a mucinous phenotype belongs to a pathway of hormonal escape in prostate cancer. Mucosecreting cells positive for MUC5AC were present in the Cowper's glands and prostatic urethral epithelium near the utriculum (Daher *et al*, 1990). Anti-MUC5AC antibodies stained the cytoplasm of rare cells in PAC120 and did not stain HID variants. MUC5B and MUC6 were detected in the mucin lakes of mucinous differentiated variants. So, these secreted mucins were independently secreted and expressed in a cell type-specific manner. Overall, MUC2, MUC5B and MUC6 were associated with mucinous morphology, and were expressed in HID prostate tumour variants. These three mucins are encoded by genes colocated in the chromosome 11p15. Their *de novo* expression seemed to be concomitant, suggesting either an amplification of this region or a deregulated expression, which lends further support to the possibility that these mucins are involved in hormonal escape. Mucin secretion may affect interactions with the extracellular environment, which could directly or indirectly influence proliferation and/or apoptosis of prostate cancer cells. Additionally, secreted mucins may directly affect tumour cell behaviour through interactions with membrane components. Indeed, a recent study revealed the role of MUC5AC, MUC1 and proteoglycans in the inhibition of E-cadherin function associated with invasiveness of HT-29 colon adenocarcinoma cell variants (Truant *et al*, 2003). We are currently investigating the link between the expression of secreted mucins, E-cadherin function and perturbations of the androgen receptor signalling pathway in hormone-independent prostate tumours.

In conclusion, the loss of hormone dependence in this prostate cancer xenograft model is marked by irreversible histological alterations, mucinous or neuroendocrine, that are associated with a constant increase in the expression of secretory MUC2, MUC5B and MUC6, which might participate in an unknown pathway of hormone escape in prostate cancer. These data point to mucinous differentiation as an important step in the acquisition of hormone independence in prostate cancer.
